# Impact of caspase-1/11, -3, -7, or IL-1*β*/IL-18 deficiency on rabies virus-induced macrophage cell death and onset of disease

**DOI:** 10.1038/cddiscovery.2017.12

**Published:** 2017-03-06

**Authors:** E Kip, F Nazé, V Suin, T Vanden Berghe, A Francart, S Lamoral, P Vandenabeele, R Beyaert, S Van Gucht, M Kalai

**Affiliations:** 1 National Reference Centre of Rabies, Viral Diseases, Communicable and Infectious Diseases, Scientific Institute of Public Health (WIV-ISP), Brussels, Belgium; 2 Inflammation Research Center, Unit of Molecular Signal Transduction in Inflammation, VIB, Ghent, Belgium; 3 Department of Biomedical Molecular Biology, Ghent University, Ghent, Belgium; 4 Inflammation Research Center, Molecular Signalling and Cell Death Unit, VIB, Ghent, Belgium; 5 Laboratory of Virology, Department of Virology, Parasitology and Immunology, Faculty of Veterinary Medicine, Ghent University, Ghent, Belgium

## Abstract

Rabies virus is a highly neurovirulent RNA virus, which causes about 59000 deaths in humans each year. Previously, we described macrophage cytotoxicity upon infection with rabies virus. Here we examined the type of cell death and the role of specific caspases in cell death and disease development upon infection with two laboratory strains of rabies virus: Challenge Virus Standard strain-11 (CVS-11) is highly neurotropic and lethal for mice, while the attenuated Evelyn–Rotnycki–Abelseth (ERA) strain has a broader cell tropism, is non-lethal and has been used as an oral vaccine for animals. Infection of Mf4/4 macrophages with both strains led to caspase-1 activation and IL-1*β* and IL-18 production, as well as activation of caspases-3, -7, -8, and -9. Moreover, absence of caspase-3, but not of caspase-1 and -11 or -7, partially inhibited virus-induced cell death of bone marrow-derived macrophages. Intranasal inoculation with CVS-11 of mice deficient for either caspase-1 and -11 or -7 or both IL-1*β* and IL-18 led to general brain infection and lethal disease similar to wild-type mice. Deficiency of caspase-3, on the other hand, significantly delayed the onset of disease, but did not prevent final lethal outcome. Interestingly, deficiency of caspase-1/11, the key executioner of pyroptosis, aggravated disease severity caused by ERA virus, whereas wild-type mice or mice deficient for either caspase-3, -7, or both IL-1*β* and IL-18 presented the typical mild symptoms associated with ERA virus. In conclusion, rabies virus infection of macrophages induces caspase-1- and caspase-3-dependent cell death. *In vivo* caspase-1/11 and caspase-3 differently affect disease development in response to infection with the attenuated ERA strain or the virulent CVS-11 strain, respectively. Inflammatory caspases seem to control attenuated rabies virus infection, while caspase-3 aggravates virulent rabies virus infection.

## Introduction

Rabies virus is a negative single-stranded RNA virus belonging to the *Genus Lyssavirus*, *Familia Rhabdoviridae*. It consists of a helicoidal nucleocapsid, surrounded by a phospholipid membrane, which contains an external glycoprotein (G) and an internal matrix protein (M).^1^ Rabies virus is a primary encephalotropic virus, which causes significant mortality worldwide and remains an important public health problem. Rabies causes about 59000 human deaths each year.^2^ Neurons and muscle cells have long been recognized as the typical target cells of rabies virus, but we showed that macrophages can also be infected productively and transmit the virus upon cell transfer *in vivo*.^3^ The neurovirulence of rabies virus is often studied in rodent models using fixed laboratory strains with different degrees of pathogenicity. The Challenge Virus Standard strain-11 (CVS-11) is a fixed laboratory virus that shows a strong neurotropism in mice and causes fatal acute encephalitis.^4,5^ Evelyn–Rotnycki–Abelseth virus (ERA) is a highly attenuated laboratory strain that was adapted to replicate in non-neuronal cells^6^ and induces an abortive, non-lethal infection of the nervous system. ERA triggers an efficient immune response and has been used as a live vaccine for immunization of wildlife.^7^ Preventive therapy for rabies is efficacious, but there is no effective therapy for patients with symptoms. A better understanding of basic mechanisms underlying the pathogenesis of rabies encephalitis, including cell death processes, is needed.^8^

Programmed cell death is a process by which cells participate in their own death in a regulated manner. Viral infections often elicit programmed cell death as part of the host defense system to cut virus infection and it is not surprising that this is targeted by pathogen-encoded cell death suppressors to modulate host cell death pathways.^9,10^ Apoptosis and pyroptosis constitute major cell death modes for elimination of infected cells and are characterized by distinct signaling pathways and morphologic changes.

Apoptosis is a non-lytic and caspase-dependent host defense pathway triggered through cell-intrinsic and cell-extrinsic factors. The extrinsic pathway is based on activation of cell death receptors, whereas the intrinsic pathway occurs through the mitochondria. Both pathways lead to activation of effector caspases, which ultimately form the apoptosome. There are 13 members of the caspase family. Caspases involved in apoptosis include caspase-2, -8, -9, -10, and -12 (the initiator caspases) and caspase-3, -6, -7, and -14 (the effector caspases).^9^ Rabies virus has been shown to induce apoptosis and caspase activation in infected cells,^11–13^ but the role of apoptosis in pathogenicity is controversial. Apoptotic neurons were detected in the brain of mice upon intracranial inoculation of virulent rabies virus.^14–16^ Jackson *et al.*^17^ suggest that there is an inverse relationship between pathogenicity and neuronal apoptosis and that neuronal apoptosis does not contribute to pathogenicity in human rabies encephalitis,^18^ even if apoptosis is detected in brains of human patients.^19^ Others reported that rabies virus can infect mouse and human lymphocytes and induce their apoptosis^20,21^ or suggested that T lymphocytes invading the brain undergo apoptosis, which may promote the neuroinvasion and spread of virulent rabies virus.^22^ A more recent study compared different strains of rabies virus (virulent street virus CNM11 and attenuated virus CTN) and detected active caspase-3 in the brain of mice infected with attenuated or virulent rabies virus.^23^ A higher rate of apoptosis was detected in brains of mice infected with the attenuated virus, with the location of apoptosis depending on the strain. Apoptosis is thus clearly induced during rabies infection but its exact contribution in rabies pathogenicity with either attenuated or virulent rabies virus remains unclear.

Pyroptosis is an inflammasome-dependent cell death mode executed following activation of inflammatory caspases caspase-1 or mouse caspase-11. The inflammatory caspases in humans include caspase-1, caspase-4, caspase-5, and caspase-12. In mice, caspase-11 is the ortholog of human caspase-4 and caspase-5.^24^ Pyroptosis releases intracellular contents, like necrosis, but also induces nuclear condensation and DNA fragmentation, like apoptosis.^25^ Canonical inflammasome activation results in procaspase-1 cleavage and activation. Distinct inflammasomes can be formed depending on the infectious agent and to date, five receptors have been established to form an inflammasome named NLRP1, NLRP3, NLRC4, AIM2, and PYRIN.^24,26^ Activated-caspase-1 is responsible for the proteolytic maturation of the inflammatory cytokines pro-IL-1*β* and pro-IL-18 in myeloid cells and neurons, which makes pyroptosis a proinflammatory cell death mode. On the other hand, the activation of a noncanonical inflammasome results in activation of procaspase-11. Caspase-11 is required for IL-1*β* release and pyroptosis in response to Gram-negative bacteria and cytosolic LPS.^27,28^ The canonical and noncanonical inflammasomes regulate release of IL-1*α* and IL-1*β* and both caspase-1 and caspase-11 can induce pyroptosis, but only caspase-1 processes preforms of IL-1*β* and IL-18 into active forms, which are secreted.^28^ Many RNA viruses, such as encephalomyocarditis virus, vesicular stomatitis virus, measles virus, hepatitis C virus, and influenza virus, have been shown to activate the NLRP3 inflammasome.^29^ Rabies virus was shown to be recognized by the NLRP3 inflammasome and to activate IL-1*β* release in murine dendritic cells.^29,30^ In that study, IL-1R-deficient mice showed an increase in rabies virus pathogenicity, but the exact contribution of inflammasome-mediated pyroptosis and IL-1*β* release in rabies pathogenicity is still unknown.

It is unclear what is the role of different caspases in rabies virus-induced cytotoxicity and whether cell death is detrimental, by contributing to pathogenicity, or beneficial, by limiting virus spread, for the host.^10^ In this study, we used specific caspase-deficient mice and macrophages to investigate the role of different caspases and cell death pathways in rabies virus infection and pathogenesis. First, we examined the type of cell death and pathways that are activated by rabies virus in an *in vitro* cell model. The spleen macrophage-derived Mf4/4 cell line was initially used because previous studies showed these cells are highly susceptible for cell death upon infection with rabies virus. The association between infection, virus production, and cell viability was examined and compared for two rabies virus strains with contrasting pathogenic properties, CVS-11 and ERA. Western-blot and immunoprecipitation were applied to study the activation of apoptotic and pyroptotic pathways in Mf4/4 macrophages. The impact of caspase-1/11, -3, or -7 deficiency on virus-induced cell death was further studied in bone marrow-derived macrophages (BMDM). Finally, we examined the impact of a deficiency in caspase-1/11, -3, -7, or IL-1*β*/IL-18 on the virological and clinical outcome of rabies virus infection in mice.

## Results

### ERA and CVS-11 cause cell death and activation of caspases in Mf4/4 macrophages

We have shown before that rabies virus can infect macrophages *in vitro*, but cell death signaling pathways in that type of cell and their impact on the development of disease were not yet explored. Here we compared the cytotoxicity of attenuated ERA and virulent CVS-11 strains toward the cell line Mf4/4. Therefore, we monitored the production of infectious rabies virus particles and cell viability up to 48 h post inoculation (hpi) of the cells with the virus.

**Figure 1:**
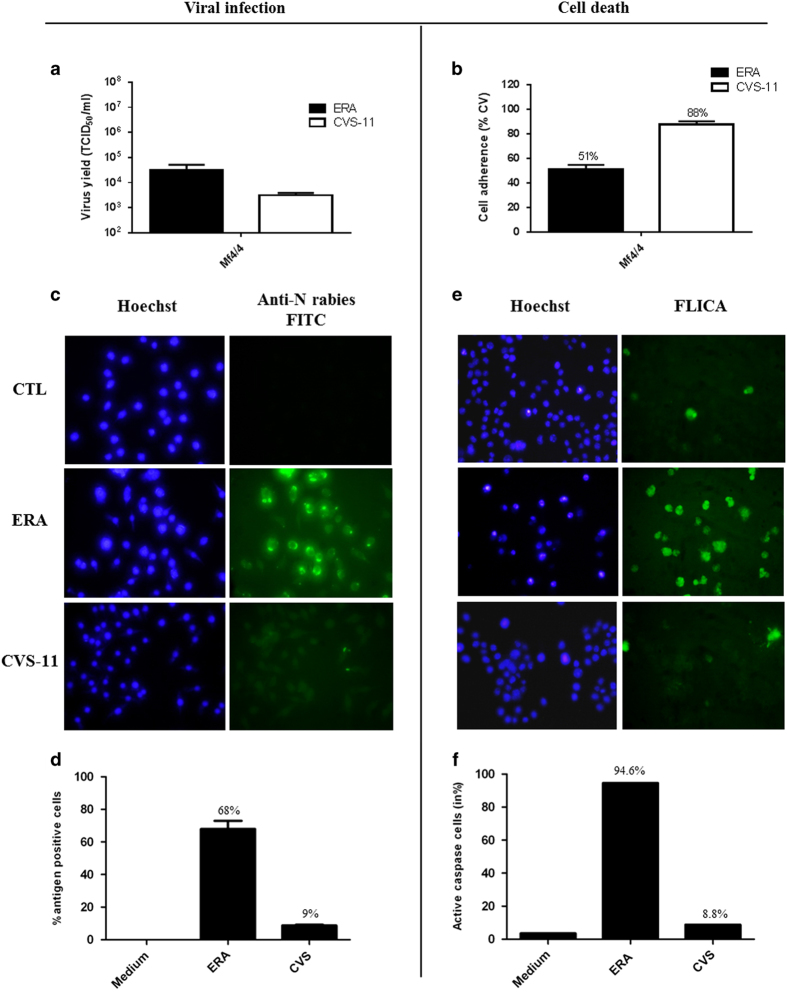
Comparison between viral load and cell death in Mf4/4 cells following infection with ERA or CVS-11 rabies virus strains. (**a**) Virus titer was measured at 48 hpi using titration in BHK-21 cells. (**b**) Cellular viability was determined using the crystal violet staining method to measure the viable adherent cells as a percentage compared to uninfected control cells. Data are representative of at least five independent experiments and are presented as mean values±S.E.M. (**c**) Fluorescence microscopy imaging of viral N protein to measure the number of infected Mf4/4. Cells were stained with Hoechst (left panel) and FITC-labeled anti-rabies virus nucleocapsid antibody (right panel). (**d**) The percentage of antigen-positive cells is expressed by: (number of FITC-anti-N cells/numbers of Hoechst-positive cells)×100. (**e**) Analysis of caspase activity in infected Mf4/4 cells at 48 h post virus inoculation. Cells were stained with Hoechst (left panel) and with the green fluorescent caspases inhibitor (FLICA) that covalently binds to the active caspases (right panel). (**f**) The percentage of cells with active caspases is expressed by: (number of FLICA-positive cells/number of Hoechst-positive cells)×100.

Infection of Mf4/4 cells with CVS-11 yielded 10^3.5^ TCID_50_/ml infectious particles, which was 10–15-fold less productive than with the attenuated ERA strain (Figure 1a). The rate of infected cells was determined using fluorescence microscopy imaging of cells stained with an antibody directed to the viral N protein (Figures 1c and d). The majority of the ERA virus-inoculated Mf4/4 cells were infected, since at least 68% expressed the viral N protein. However, only 9% of the macrophages inoculated with the CVS-11 were fluorescent for viral N protein (Figures 1c and d). The percentage of infected cells and production of infectious rabies virus particles correlated with the rate of cell death, as assessed by measuring cell adherence. A strong decrease of macrophage cell adherence was observed upon infection with ERA (Figure 1b). Only 51% of Mf4/4 remained adherent 48 hpi, which is significantly less than with the virulent strain (88%) (*P*<0.001, two-way ANOVA).

In parallel, Mf4/4 cell death induced by rabies virus was analysed by the binding of the intracellular green fluorescent caspase inhibitor (FLICA) to active caspases (Figures 1e and f). Caspase activation was seen in 94,6% of ERA-inoculated cells, while infection with CVS-11 induced activation of caspases only in 8,8% of the cells, which corresponds with the lower infection rate of CVS-11 (Figures 1e and f). Mock-infected macrophages showed no fluorescence in the presence of FLICA. These data suggest that caspase activation is involved in rabies virus-induced Mf4/4 cell death and that ERA infects and replicates more efficiently in macrophages than CVS-11. Moreover, both strains induce cell death,^3^ but since more macrophages become infected with ERA than with CVS-11, caspase activation is more obvious with the former.

### Activation of signaling pathways for apoptosis and pyroptosis in rabies virus-infected Mf4/4

We analysed two forms of cell death requiring caspase activation, namely apoptosis and pyroptosis, in rabies virus-infected macrophages. Since Mf4/4 are highly susceptible to infection with ERA, we analysed lysates of ERA virus-infected Mf4/4 by western blotting at 24 and 48 hpi using antibodies against different caspases and the pro-apoptotic peptide truncated Bid (tBid). Specific antibodies against active IL-1*β* and caspase-1 were used as pyroptosis markers.

**Figure 2:**
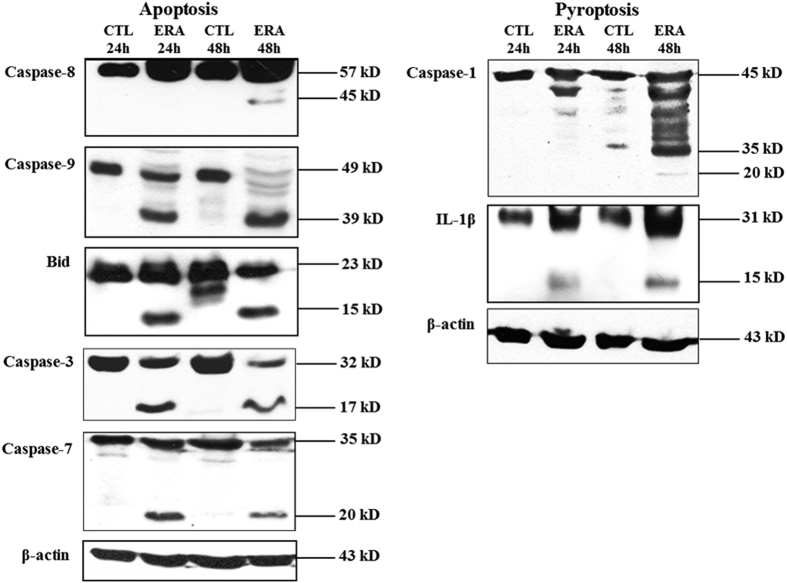
Activation of signaling pathways of apoptosis and pyroptosis in rabies virus-infected Mf4/4. Analysis of the proteolytic activation of caspases-1, -3, -7, -8, and -9, IL-1*β* and Bid in MF4/4 cells inoculated by ERA. Western blots were performed using antibodies specific for the active and inactive forms of these proteins. Upon activation, the protein is cleaved and appears as a smaller band representing the active protein on the blot. Total cellular lysates were prepared 24 and 48 h after virus inoculation. Uninfected Mf4/4 were prepared in parallel to each condition as negative controls (CTL). An anti-*β*-actin antibody was used to verify that equal amounts of protein were loaded. Data are representative of at least three independent experiments.

Analysis of cell lysates of ERA virus-infected Mf4/4 indicated that cleaved active caspase-3, -7, and -9 and tBid were detected 24 hpi, whereas proteolytic cleavage of initiator caspase-8 and the 20 kDa fragment of cleaved and active caspase-1 were detected at 48 hpi (Figure 2). However, the 15 kDa fragment corresponding to the active mature form of IL-1*β* could be immunoprecipitated from the culture supernatant of infected Mf4/4 already at 24 hpi. The cleavage of inactive 31 kDa precursor IL-1*β* to the active mature cytokine requires active caspase-1, which suggests that caspase-1 was already active at 24 hpi. These observations indicate that infection of Mf4/4 by ERA involves the proteolytic activation of several caspases contributing to cell death.

### Caspase-3 deficiency protects against rabies virus-induced apoptosis in BMDM

**Figure 3:**
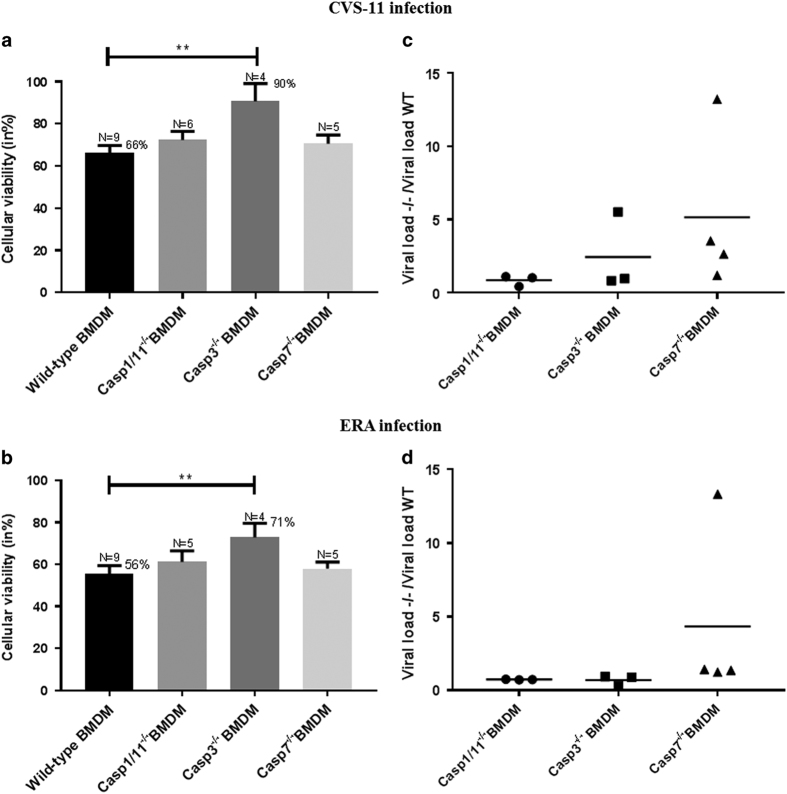
Cellular viability and viral load of caspases-1/11, -3, or -7-deficient primary BMDMs inoculated by CVS-11 (**a**, **c**) or ERA (**b**, **d**) virus. (**a**, **b**) Cellular viability was determined at 48 hpi using the crystal violet staining method to measure the percentage of viable adherent cells. *P*-value was calculated using an unpaired *t*-test (each group of deficient BMDM compared to the WT group) and was indicated as follows: *P*-value<0.01 (**). A protector effect on the viability of the cells was observed in caspase-3^−/−^ macrophages inoculated with ERA or CVS-11. Data were pooled and are representative of at least four independent experiments. They are presented as mean values±S.E.M. (**c** and **d**) Viral load was determined 48 hpi by RT-qPCR following CVS-11 or ERA infection. Results are presented as a ratio compared to the WT cells. Each point represents one independent experiment.

BMDM from wild-type (WT) and deficient mice (caspase-1/11, -3, or -7 deficient) were used to assess whether the main effectors of pyroptosis (caspase-1 and -11) and apoptosis (caspase-3 and -7) are involved in rabies virus-induced cell death. After isolation, cells were infected with either ERA or CVS-11 and cell adherence was assessed by crystal violet staining as a measure for cell viability 48 hpi. Interestingly, a significant protective effect on BMDM viability was observed in caspase-3^−/−^ macrophages infected by ERA or CVS-11 virus (Figures 3a and b). Indeed, an average of 90% viability was observed in caspase-3^−/−^ BMDM infected with CVS-11 compared to an average of 66% in WT cells. For ERA virus, an average of 71% viability was observed in caspase-3^−/−^ BMDM, compared to an average of 56% in WT cells. In caspase-1/11^−/−^ or caspase-7^−/−^ BMDM, no significant protective effect against cell death was observed. In parallel, we also examined the effect of caspase-1/11, -3, or -7 deficiencies on rabies virus replication in macrophages. WT and deficient BMDM were infected with ERA and CVS-11 and the viral load was determined by RT-qPCR. Results obtained with caspase-7^−/−^ or caspase-3^−/−^ in comparison to WT BMDM following 48 hpi tended to be highly variable (Figures 3c and d). Nevertheless, the mean viral load KO/WT ratios obtained with ERA or CVS-11 suggest that caspases-7 may affect rabies virus replication. In contrast, the variability observed in KO/WT viral load ratios obtained with caspases-1^−/−^ macrophages after ERA or CVS infection was low, with means being just below 1.0, suggesting no or low impact for the caspase on viral replication.

### Deficiency of caspase-3, but not caspase-1/11, -7, or IL-1*β*/ IL-18 delays CVS-11-induced disease in mice

The impact of cell death on rabies pathogenicity is controversial. To better understand the impact of apoptosis and pyroptosis *in vivo* during infection with a virulent rabies strain, we studied the impact of caspase-1/11, -3, or -7 deficiency in mice on rabies virus-induced morbidity and mortality following intranasal inoculation with CVS-11. Since the production of active IL-1*β* and IL-18 depends on caspase-1 activity, we also analysed the impact of IL-1*β*/IL-18 double deficiency in the same experimental set-up. Neutralizing antibody concentrations in serum and viral load in the brain were determined at the moment of killing.

**Figure 4:**
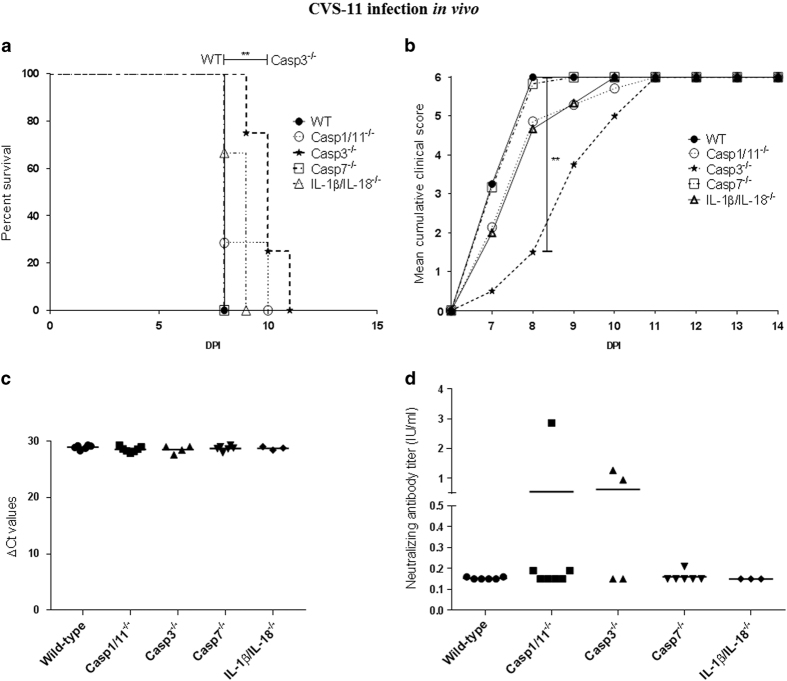
Impact of a deficiency in caspase-1/11, -3, or -7 or IL-1*β*/IL-18 in mice on rabies disease progression, mortality, brain viral load, and neutralizing antibody response upon infection with a virulent strain of rabies. WT (*n*=6), caspase-1/11^−/−^ mice (*n*=7), caspase-3^−/−^ mice (*n*=4), caspase-7^−/−^ mice (*n*=6), and IL-1*β*/IL-18^−/−^ mice (*n*=3) were inoculated intranasally with the virulent CVS-11 virus (10^2.5^ TCID_50_/mouse). Disease progression and mortality within each group was evaluated by mean clinical score per mouse (**b**) and survival curves (**a**), respectively. *P*-value was calculated using a log-rank test (survival curves) and an ANOVA test followed by Bonferroni multiple comparison post-test (mean score per mouse) and was indicated as follows: *P*-value <0.05 (*); *P*-value <0.01 (**). (**a**) Survival curves of WT mice and caspase-3-deficient mice were significantly different (**). (**b**) The mean clinical score per mouse was significantly different between WT and caspase-3^−/−^ mice at 8 DPI. Caspase-3 deficiency delayed CVS-11-related disease, but had no effect on final lethal outcome. (**c**) Brain tissue was sampled upon peak clinical score and viral load measured by RT-qPCR. *P*-value was calculated with an ANOVA test followed by Bonferroni multiple comparison post-test. No significant difference could be observed between WT mice and deficient mice. (**d**) The neutralizing antibody titer was evaluated by rapid fluorescent focus inhibition test and expressed in IU/ml. *P*-value was calculated by an ANOVA test followed by Bonferroni multiple comparison post-test. Most mice were unable to mount an antibody response (>0.5 IU/ml) prior to killing. No significant difference was observed between WT and deficient mice. Two out of four caspase-3^−/−^ mice had developed antibody titers >0.5 IU/ml, which might be because they survived long enough to allow an antibody response.

WT and deficient mice inoculated with CVS-11 strain developed severe disease, beginning with weight loss and appearance of a hunched back, followed by motoric incoordination, a wasp waist and in the last stage of the disease depression and paralysis of the hind legs. All mice reached the clinical end point, requiring killing, about 1–3 days after the appearance of the first disease symptoms. For caspase-3^−/−^ mice, the onset of symptoms was delayed by 2 days, compared to their corresponding WT mice (Figure 4b). At 8 DPI, most of the WT, caspase-1/11, -7, and IL1*β*/IL-18-deficient mice had reached the maximal clinical score, while the caspase-3^−/−^ mice were only starting to show the initial symptoms of the disease (*P*<0.01). Overall, mortality was 100% in WT and caspase-deficient mice, but the median survival time was significantly longer for caspase-3^−/−^ mice (*P*<0.01). This was not the case for caspase-1, -7, and IL-1*β*/IL-18-deficient mice. The median survival time was 8 DPI in WT mice, caspase-1/11, -7, and IL1*β*/IL-18-deficient mice and 10–11 DPI in caspase-3^−/−^ mice (Figure 4a). We have also determined the effect of caspase-1, -3, -7, or IL1*β*/IL-18 deficiency on the final cerebral virus load at the moment of killing (=peak of disease), but there was no significant difference (Figure 4c). At the moment of killing (8–11 DPI), none of the mice had developed antibody levels above the protective threshold of 0.5 IU/ml (Figure 4d). In conclusion, these results indicate that apoptosis through caspase-3 promotes early onset of disease upon infection with a virulent strain of rabies virus, but has no effect on final viral load in the brain or on final disease outcome.

### Deficiency of caspase-1/11, but not caspase-3, -7, or IL-1*β*/ IL-18 increases ERA-induced morbidity in mice

**Figure 5:**
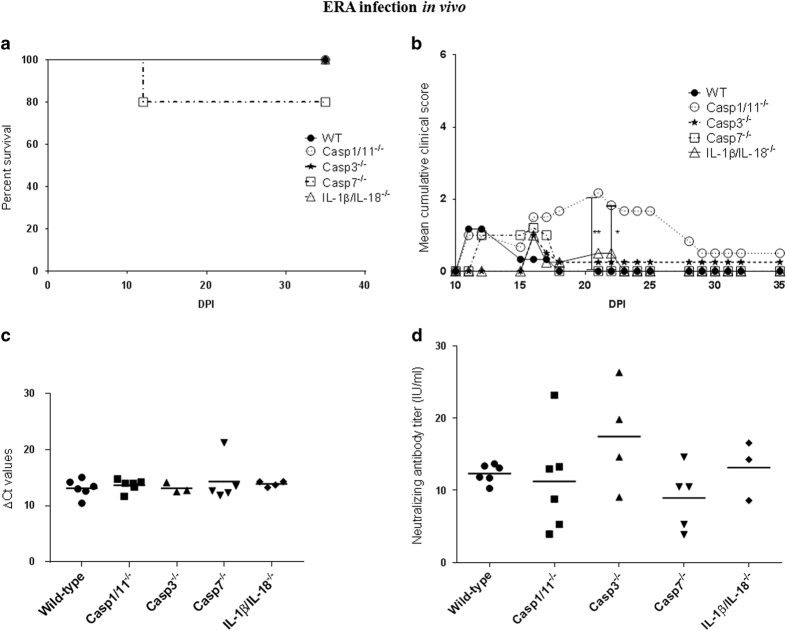
Impact of a deficiency in caspase-1/11, -3, or -7 or IL-1*β*/IL-18 in mice on rabies disease progression, mortality, brain viral load, and neutralizing antibody response upon infection with an attenuated strain of rabies. WT (*n*=6), caspase-1/11^−/−^ mice (*n*=6), caspase-3^−/−^ mice (*n*=4), caspase-7^−/−^ mice (*n*=5), and IL-1*β*/IL-18^−/−^ mice (*n*=4) were inoculated intranasally with the attenuated ERA virus (10^5^ TCID_50_/mouse). Disease progression and mortality within each group was evaluated by mean clinical score per mouse (**b**) and survival curves (**a**), respectively. *P*-value was calculated using a log-rank test (survival curves) and an ANOVA test followed by Bonferroni multiple comparison post-test (mean score per mouse) and was indicated as follows: *P*-value <0.05 (*); *P*-value <0.01 (**). (**a**) All mice survived the infection except for one caspase-7^−/−^ mouse. (**b**) The mean clinical score per mouse was significantly different between WT and caspase-1^−/−^ inoculated with ERA virus at 21 and 20 DPI (**c**). Brain tissue was sampled at 35 DPI (except for one mouse) and viral load measured by RT-qPCR. *P*-value was calculated with an ANOVA test followed by Bonferroni multiple comparison post-test. No significant difference could be observed between WT mice and deficient mice. (**d**) The neutralizing antibody titer was evaluated by rapid fluorescent focus inhibition test and expressed in IU/ml. *P*-value was calculated by an ANOVA test followed by Bonferroni multiple comparison post-test. No significant difference was observed between WT and deficient mice, but antibody titers seemed higher in caspase-3^−/−^ mice.

ERA virus is not lethal and induces only minor symptoms in immunocompetent WT mice. The impact of cell death following infection with an attenuated rabies strain has been poorly investigated *in vivo*. We found that intranasal inoculation of ERA virus in WT, caspase-3, -7, or IL1*β*/IL-18^−/−^ mice led to the development of minor symptoms such as weight loss or motoric incoordination from 11–18 DPI. In contrast, caspase-1/11^−/−^ mice developed more severe symptoms, such as hunched back and paralysis, which lasted for a longer period, with some symptoms still present at 35 DPI, the end of the observation period. At 20 and 21 DPI, clinical scores were significantly higher for caspase-1/11^−/−^ compared to other mice (Figure 5b). Interestingly, such an ERA-induced morbidity was not observed with IL-1*β*/IL-18^−/−^ mice, suggesting that the impact of caspase-1/11^−/−^ was not due to lack of these cytokines. No mortality was observed in WT and caspase-1/11, -3, -7, or IL-1*β*/IL-18-deficient mice inoculated with ERA.

We next determined if caspase or IL-1*β*/IL-18 deficiency had an impact on viral load in the brain and neutralizing antibody production. ERA virus-inoculated mice were killed at 35 DPI. At that stage, viral RNA was still detectable in the brain, with no significant differences between deficient and WT mice (Figure 5c). This persistence of ERA virus RNA was not linked to disease, as most symptoms had disappeared before 35 DPI. All mice developed a strong antibody response, largely above 0.50 IU/ml, and there was no significant difference in antibody titers between WT and deficient mice (Figure 5d). In conclusion, our results suggest that caspase-1 or caspase-11 controls attenuated ERA strain infection and disease within the central nervous system.

## Discussion

Apoptosis and pyroptosis can be induced by a wide variety of stimuli, including viral infection. In this work, we analysed the impact of key mediators of apoptosis (caspase-3 and -7) and pyroptosis (caspase-1 and -11) on rabies virus production, cell death, morbidity, and mortality. Cells of the immune system seem prone to undergo cell death upon infection with rabies virus.^20^ This seems particularly the case for the ERA-attenuated strain, which appears also better fit than CVS-11 to replicate in these cells. For the *in vitro* studies, macrophages were used as the infection model, since these cells are very susceptible to infection,^31–33^ especially with the ERA strain, and undergo distinguishable cell death upon infection. Infection of macrophages and their resulting cell death was rabies strain-dependent: we observed significantly higher infection rates, accompanied by a strong apoptotic response, with the ERA strain than with the CVS-11 strain. Interestingly, Thoulouze *et al.*^20^ reported that *in vitro* infection of a human lymphoblastoid Jurkat T-cell line with ERA, but not with CVS-11, causes apoptosis. They suggested that the improved capacity of the ERA strain to stimulate the immune system could be paradoxically linked to its ability to induce apoptosis in immune system cells. Apoptosis of infected cells may augment the presentation of viral antigen and then efficiently activate the immune system.

Another study, conducted on the brain of deceased rabies patients, demonstrated immunostained cleaved caspase-3 in microglial cells, which are from the macrophage lineage.^18^ In our study, we demonstrate that caspases-1/11, -3, -7, -8, and -9, IL-1*β* and the Bid factor are activated in Mf4/4 cells upon infection with ERA. They are also activated after infection with CVS-11, but to a lesser extent which is probably linked to the lower rate of infection of Mf4/4 cells. By western blot analysis, we found that caspase-9 and Bid factor were activated earlier than caspase-8, suggesting that the apoptotic process is initiated by the activation of the apoptosome via the mitochondrial pathway and Bid factor proteolysis.^34^ The IL-1*β* activation demonstrates the involvement of caspase-1 in the inflammatory process induced by the ERA strain in Mf4/4.

Our results on primary BMDM suggest a predominant role of caspase-3 in cell death induced by ERA and CVS-11 strains in macrophages, while caspase-1/11 or -7 does not seem to be necessary. *In vivo*, apoptotic (cytochrome c, caspase-3) and pyroptotic (caspase-1, IL-1*β*) genes were shown to be upregulated following rabies virus infection.^35^ In our study, the onset of disease upon rabies virus infection was significantly delayed in caspase-3^−/−^ mice, compared to WT, IL-1*β*/IL-18, caspase-1/11, and caspase-7-deficient mice. This might be explained by an involvement of neuronal apoptosis in the cerebral dysfunction associated with rabies disease. Indeed, it was shown that CVS-11 produces neuronal apoptosis in the brain of mice after intracerebral inoculation, without implication of the adaptive immune system, as demonstrated in nude and Rag1 mice;^36,37^ moreover, we observed immunostaining for cleaved caspase-3 in neuronal cells of CVS-11-infected mice in our laboratory. In another study, infection of bax-deficient mice with CVS-11 reduced apoptosis of neurons from the dentate gyrus and cortical neurons, but still yielded 100% mortality.^38^ Moreover, the apoptotic cell death of neurons is a mechanism known to be implicated in other types of infectious brain disease, such as reovirus-induced encephalitis.^39^ Other studies showed no apoptosis in neurons of rabies virus-infected mice infected intramuscularly by CVS-11, but apoptosis was detected in microglial cells and T lymphocytes, whereas neurons died rather by necrosis.^40^ In another study, apoptosis was not detected in the brain of experimentally infected mice infected with a street rabies virus (silver-haired bat rabies virus), although rabies virus antigens were distributed widely within the CNS.^41^ The variable results in literature could be related to differences in virus strain, route of inoculation, or the strain of laboratory animals used.^15,23^ Viral loads were similar in all deficient and WT mice, including caspase-3^−/−^ mice, despite the delay in disease observed. However, it is important to mention that samples were only taken at the time of killing, when all mice had developed a similar level of disease. Analysis of the viral load at comparable and earlier time points should be performed for more firm conclusions.

There is good evidence that pyroptosis can benefit the host during infection but if too many host cells undergo pyroptosis, detrimental effects may occur due to the release of inflammatory cytosolic mediators or the depletion of host cells, which highlights the notion of good *versus* bad inflammation. Previous studies have shown that increased IL-1*β* mRNA levels were found in brains of paralytic rabid dogs during early stages of infection^42^ and IL-1*β* release and Nlrp3 inflammasome activation were demonstrated in murine dendritic cells infected by rabies virus.^30^ Still, the impact of pyroptosis in rabies pathogenicity is not well established. We were not able to prove the involvement of caspase-1 or -11 or IL-1*β*/IL-18 on CVS-11-induced morbidity or mortality. On the other hand, infection of caspase-1/11^−/−^ mice with the ERA strain showed a worsening of symptoms in comparison to WT mice. Inflammation is thought to promote the viral clearance in rabies virus infection, especially when the strain is attenuated.^43,44^ So, the capacity of caspase-1 to take part in the inflammatory process by cleaving IL-1*β* could have been a reasonable explanation. However, in contrast with caspase-1/11^−/−^ mice, inoculation of IL-1*β*/IL-18^−/−^ mice with the ERA strain induced no worsening of symptoms in comparison with WT mice. The higher morbidity observed in caspase-1/11^−/−^ mice is thus probably not linked to IL-1*β*/IL-18 activation and secretion. It is important to note that the caspase-1/11^−/−^ mice were initially considered as caspase-1^−/−^, but the double deficiency with caspase-11 was later recognized.^28^ Thus, the results observed in our study might also involve caspase-11, which is also implicated in the pyroptosis pathway.

In conclusion, in this study, infection of macrophages was shown to lead to apoptotic and pyroptotic cell death. *In vivo*, apoptosis mediated through caspase-3 seems more detrimental than protective for the host upon infection with a virulent strain (CVS-11). Blocking apoptosis with caspase-3 inhibitors might therefore have beneficial effect on disease. In contrast, during attenuated rabies strain infection, pyroptosis mediated by caspase-1 and/or caspase-11 plays a role in limiting the disease caused by an attenuated rabies virus strain (ERA).

## Materials and Methods

### Virus

The highly virulent neurotropic CVS-11 strain and the attenuated ERA virus, used as an oral vaccine for immunization of wild life, were obtained from the American Type Culture Collection (references: VR959 and VR322, respectively).^45^ Virus stocks were grown in baby hamster kidney (BHK)-21 cells. The lysates of infected cell cultures were centrifuged at 20 000×*g* for 20 min at 4 °C and the infectious dose was titrated in BHK-21 cells (see below).

### Antibodies and reagents

Crystal violet was purchased from UCB (Brussels, Belgium). FITC-coupled anti-nucleocapsid rabbit antibody was from Bio-Rad Laboratories (Hercules, CA, USA). Horseradish peroxydase (HRP)-linked anti-rat IgG, mouse-specific anti-caspase-8, -9, cleaved caspase-7 and -3 polyclonal antibodies were from BIOKE (Leiden, The Netherlands). Polyclonal anti-mouse IL-1*β*/IL-1F2 antibody, monoclonal anti-mouse BID, and monoclonal anti-mouse IL-1*β* were from R&D systems (Minneapolis, MN, USA). Mouse-specific anti-caspase-1 antibody was made in-house as described before.^46^ The anti-rabies virus glycoprotein mouse monoclonal antibody and HRP-linked anti-goat IgG were from Santa Cruz (Santa Cruz, CA, USA). HRP-linked anti-mouse and anti-rabbit antibody were from GE Healthcare (Waukesha, WI, USA). HRP-conjugated anti-*β*-actin antibody was from ICN Biomedicals (Irvine, CA, USA).

### Cell culture and cellular infection

Mf4/4 is an immortalized cell line of spleen macrophages derived from C57BL/6 mice.^47^ BHK-21 are hamster kidney cells that are routinely used to propagate laboratory strains of rabies virus. Mf4/4 were grown at 37 °C and 5% CO_2_ in RPMI-1640 (Lonza, Verviers, Belgium) supplemented with 10% fetal bovine serum, 1 mM sodium pyruvate, 100 U/ml of penicillin, and 100 *μ*g/ml of streptomycin. BHK-21 were grown in DMEM (Gibco, Paisley, UK) supplemented with 10% fetal bovine serum, 100 U/ml of penicillin, 100 *μ*g/ml of streptomycin, and 0.25 *μ*g/ml of amphotericin B.

BMDMs were isolated from femurs and tibias of 6–12 weeks old C57BL/6 mice. Bone marrow was flushed from the bones with RPMI-1640 and following centrifugation, cells were suspended in RPMI-1640 supplemented with 20 ng/ml macrophage-colony stimulating factor (Peprotech, Rocky Hill, CT, USA) and seeded in a T75 flask. Non-adherent cells were washed away 4 days later. After 6 days, adherent differentiated macrophages were collected and reseeded in plates. Macrophage purity was ~98.8% as assessed by flow cytometry using the phycoerythrin-conjugated monoclonal antibody BM8 anti-F4/80 macrophage marker (eBioscience, Hartfield, UK).

For cellular infection, Mf4/4 were seeded and infected 24 h later, with the CVS-11 or ERA strains at a multiplicity of infection of 1. BMDMs were seeded and infected 2 h later with rabies virus at multiplicity of infection 1 in the presence of macrophage-colony stimulating factor. The cells were washed 3 h later two times with medium. At the indicated time following infection, cells were washed and fixed with methanol for 5 min to measure cell adherence as an indicator for cell viability. Alternatively, cells were frozen and used for viral titration or collected for RT-qPCR analysis.

### Measurement of cell survival

Adherence of Mf4/4 or BMDM was used as a measure for cell survival and was monitored 48 hpi by crystal violet staining. Adherent cells fixed with methanol were stained with 0.1% crystal violet for 10 min and washed twice with PBS. Crystal violet-stained cells were solubilized with a 1% sodium dodecyl sulfate (SDS) and absorbance at 595 nm was measured with a spectrophotometer (Model 680 microplate reader, BioRad, Hercules, CA, USA). Cell survival percentage was calculated using the following equation: 100%×(A595/655 treated cells—A595/655 medium)/(A595/655 untreated cells—A595/655 medium). Experiments were carried out in triplicate.

### Virus titration

Cells and medium underwent three consecutive freeze-thaw cycles at −80 °C. Supernatant and cells were collected and infectious rabies virus particles were titrated in BHK-21. Results were expressed in 50% tissue culture infective dose (TCID_50_)/ml. Virus titration was performed according to the Manual of Diagnostic Tests and Vaccines for Terrestrial Animals (Office International des Epizooties, 2008).

### Anti-nucleocapsid rabies virus staining

Mf4/4 cells were infected with ERA virus or CVS-11 as described above. At 48 hpi, cells were fixed with 75% acetone for 30 min at room temperature and incubated with FITC-coupled anti-rabies virus nucleocapsid rabbit antibody for 30 min at 37 °C. Next, a DNA Hoechst staining was performed for 5 min at 37 °C. Cells were washed with distilled water and the percentage of rabies virus-infected cells was determined using fluorescence microscopy (Nikon eclipse TS100).

### Cellular caspase activation assay

Mf4/4 cells were infected with CVS-11 or ERA virus in 24-well plates. At 48 hpi, cells were incubated with the green fluorescent caspases inhibitor (FLICA) according to the manufacturer’s instructions (apoptosis detection kit caspases assay, Eurobio-Abcys, Les Ulis, France). After washing steps, the percentage of activated caspase-positive cells was determined using fluorescence microscopy (Nikon eclipse TS100).

### Western blotting and immunoprecipitation

Mf4/4 cells were infected with ERA virus at a multiplicity of infection of 1, as described above. Mf4/4 cells inoculated with medium were used as a control. Cells and media were collected at 24 and 48 h post virus inoculation. Cells were centrifuged at 2500 × g, supernatant was collected for immunoprecipitation or discarded as indicated. The pellet was resuspended and boiled for 10 min in sample buffer containing: 6% SDS, 1.4 M *β*-mercaptoethanol, 20% glycerol, 0.01% (w/v) bromophenol blue, and 125 mM Tris-HCl, pH 6.8.

For IL-1*β* immunoprecipitation, collected supernatant media was supplemented with 10% lysis buffer (50 mM Hepes pH 7.6, 200 mM NaCl, 0.1% de NP40, and 5 mM EDTA), a protease inhibitors tablet (Roche, Basel, Switzerland) and 2 *μ*g of monoclonal anti-mouse IL-1*β*. Following 24 h incubation on a turning wheel at 4 °C, media were incubated with 20 *μ*l resuspended protein G-sepharose beads (Amersham Bioscience, Freiburg, Germany) at room temperature under gentle agitation. Following 3 h of incubation, sepharose beads were washed four times in lysis buffer and then resuspended and boiled for 5 min in sample buffer. Protein extracts and immunoprecipitation products were fractionated by SDS-PAGE and transferred to nitrocellulose membranes. Blocking, antibody incubation steps, and washing of the membrane were performed in PBS supplemented with 3% skimmed milk and 0.05% Tween 20. Blots were incubated with the indicated primary antibody overnight. Membranes were consequently incubated for 1 h with HRP-conjugated secondary antibodies to mouse, rabbit, rat, or goat immunoglobulin according to needs. Immunoreactive proteins were visualized using the chemiluminescence kit ‘Immobilon Western Chemiluminescent HRP Substrate’ (Millipore, Billerica, MA, USA) and signals were captured by exposure to film (GE Healthcare, Little Chalfont, UK).

### RT-qPCR analysis

RNA extraction was performed using RNeasy Mini kit (Qiagen, Hilden, Germany) according to the manufacturer’s instructions. Reverse transcription and q-PCR were performed as described in Rosseels *et al.*^48^ Two primers (forward and reverse, sequences are accessible on demand; Eurogentec, Seraing, Belgium) located in the nucleoprotein N genome region were used for the detection of ERA and CVS-11 strains. The cellular housekeeping gene, GAPDH, was used for normalization. All samples were analyzed in duplicates. Amplification was performed on an iCycler iQ from BioRad in a 96-well optical plate format, using the following program: 2 min at 95 °C followed by 45 cycles of 20 s at 95 °C and 30 s at 62 °C. A melting curve analysis was performed in order to verify the specificity of amplicons. Quantification of gene expression was performed using the comparative delta Ct method: delta cycle thresholds (ΔCt) values were calculated using the following formula: ΔCt=Ct_ref_−Ct_sample_, with Ct_ref_ equal to 45, which is the number of cycles of this qPCR program.

### Mice

All mice were bred under specific pathogen-free conditions, and in all experiments sex- and age-matched animals were used. Conditional caspase-7^fl/fl^ were generated on a mixed 129S6/Swiss background and back-crossed for 10 generations to the C57BL/6J background before crossing with Sox2Cre mice to generate caspase-7^−/−^ mice.^49^ Caspase-1/11^−/−^, caspase-3^−/−^, and IL1*β*/IL18^−/−^ mice on a C57BL/6J background were kindly provided by Dr. R Flavell (Howard Hughes Medical Institute, Chevy Chase, MD, USA), Dr. T Mak (The Campbell Family Cancer Research Institute, Toronto, Canada), and A Zychlinsky (Max Planck Institute, Berlin, Germany), respectively. Caspase-1/11^−/−^ mice were initially considered as caspase-1^−/−^, but the double deficiency with caspase-11 was later recognized.^28^ Mice were housed in temperature-controlled, air-conditioned facilities with 14/10-h light/dark cycles and food and water ad libitum, and used at the age of 8–12 weeks. All experimental procedures were approved by the Ethical Committee of the IPH and the Veterinary and Agrochemical Research Centre (VAR) (advice nr. 060217-03). Mice were inoculated by intranasal inoculation as described in Rosseels *et al*.,^48^ with 10^2.5^ TCID_50_ of CVS-11 virus or 10^5^ TCID_50_ of ERA virus.

### Clinical follow-up and sampling

Mice were observed daily for signs of disease throughout the experiment until 35 days post inoculation (DPI). Disease signs were scored as follows: no signs=0, >20% weight loss=1, depression=1, hunched back=1, wasp waist=1, incoordination=1, and paralysis of hind legs=1. Mice that reached a score of 6 were killed by cervical dislocation. A cumulative daily clinical score per mouse was obtained by adding the scores for each parameter. Disease progression was represented by a curve of the relative cumulative score (scores of all mice of a group/number of mice of this group). Killed mice are included in the curve by adding the score 6 for each killed mouse. Mice with no apparent rabies disease signs were killed 35 DPI. Blood and brain tissue from each mouse were sampled at the time of killing for antibody measurement by Rapid Fluorescent Focus Inhibition Test and viral load determination by RT-qPCR analysis, respectively.

### Titration of neutralizing antibody by rapid fluorescent focus inhibition test

Neutralizing antibodies were titrated with the rapid fluorescent focus inhibition test according to the Manual of Diagnostic Tests and Vaccines for Terrestrial Animals (Office International des Epizooties, 2008). Neutralizing antibody titers are expressed as international units (IU)/ml in reference to ‘The Second International standard for Anti-rabies Immunoglobulin’, purchased from the United Kingdom National Institute for Biological Standards and Control. A serum titer of 0.5 IU/ml is considered protective.

### Statistical analysis

Statistical analysis was performed using a student *t*-test or one-way ANOVA followed by Bonferroni’s multiple comparison post-test in GraphPad Prism 6. Log-rank test was used to analyse Kaplan–Meier survival curves in GraphPad Prism 6.
